# Brain Interventions, Moral Responsibility, and Control over One’s Mental Life

**DOI:** 10.1007/s12152-019-09414-7

**Published:** 2019-06-14

**Authors:** Gabriel De Marco

**Affiliations:** The Oxford Uehiro Centre for Practical Ethics, Suite 8, Littlegate House 16/17 St Ebbe’s Street, Oxford, OX1 1PT UK

**Keywords:** Brain interventions, Moral responsibility, Historicism, Manipulation

## Abstract

In the theoretical literature on moral responsibility, one sometimes comes across cases of manipulated agents. In cases of this type, the agent is a victim of wholesale manipulation, involving the implantation of various pro-attitudes (desires, values, etc.) along with the deletion of competing pro-attitudes. As a result of this manipulation, the agent ends up performing some action unlike any that she would have performed were it not for the manipulation. These sorts of cases are sometimes thought to motivate historical views of responsibility, on which the agent’s past is relevant to whether she is responsible for a specific action. In a recent paper, Daniel Sharp and David Wasserman bring these theoretical discussions on moral responsibility to bear on practical issues regarding neurological modifications of individuals. After proposing and arguing for a historical view, Sharp and Wasserman offer some insight into how such a view may help us in determining the responsibility of subjects who have undergone Deep Brain Stimulation. This paper aims to join this discussion, by arguing that the correct historical view to be applied will also appeal to the agent’s control over her mental life and the fact that this was bypassed. I conclude with some brief comments on the practical implications of such a historical view.

## Introduction

One type of case commonly discussed in the moral responsibility literature is a case of a manipulated agent. In cases of this type, the agent is a victim of wholesale manipulation, involving the implantation of various pro-attitudes (desires, values, etc.) along with the deletion of competing pro-attitudes. As a result of this manipulation, the agent ends up performing some action unlike any that she would have performed were it not for the manipulation. Suppose, for example, that Beth, the sweetest person you know, is the victim of such manipulation. While Beth is sleeping, a malevolent neurosurgeon implants in her many of the pro-attitudes held by Chuck, a man who has, repeatedly and without remorse, literally gotten away with murder. When she awakes, Beth is confronted with a desire to kill her neighbor, whom she has merely found unpleasant in the past. Although she is surprised by this desire, she correctly judges that it is in line with her system of values (the relevant parts of which were implanted as well), endorses it, and acts on it [1].

Although there are variations on manipulation cases and their purposes, they are generally thought to present a challenge for some views of moral responsibility, typically called *non-historical views*. On these types of views, what matters for responsibility is the state of the agent at, or just before, the time of action, without concern for the history of either the agent or the mental states that produced the action. Given that at the time of action (and shortly before), the manipulated agent’s mental states were related to each other in the purportedly correct way, and they produced the action in the purportedly correct way, non-historical views would tell us that these agents are as responsible for the actions that are the result of this manipulation as a normal agent would be. Yet this verdict is counterintuitive, and has led some to defend so-called historical views, which include a component requiring that the agent have had (or not have had) a certain type of history.

These sorts of wholesale manipulation are not likely to be within our grasp using currently available technologies (or those that will be available in the near future), yet some available (and soon-to-be-available) technologies may raise some related – and usually less extreme – worries.^1^ For instance, one might worry that some of the problematic features identified by historical views are present in cases involving these technologies, and that such features, although they may not fully undermine the subject’s responsibility for some later actions, may mitigate it. Historical views of moral responsibility can help guide our thinking on these less extreme versions of neurological intervention and their potential effects on an agent’s moral responsibility for actions that are the result of them. In a recent paper, Daniel Sharp and David Wasserman (S&W) undertake this project, providing their own partial historical view of moral responsibility and suggesting ways that it may apply to more realistic cases of interventions, with a specific focus on Deep Brain Stimulation (DBS) [2]. Some discussions of DBS have recently been criticized for seemingly overstating the risks of this technology, or the frequency with which various side-effects occur [3] (though see [4, 5]). I do not wish to overstate these risks here. DBS provides a useful example of a real technology that can yield these results. As I discuss later on, the views under consideration here have a much wider scope of applicability.

This paper is intended as a contribution to the project that S&W undertake. In this paper it is argued that the correct historical view will need to account for something that S&W’s view does not: an agent’s control over her mental life. I begin by briefly presenting the relevant parts of the dialectic in the moral responsibility literature. Next, I present S&W’s view, as well as a counterexample to it. After this, I present an alternative partial view that can handle this sort of case. In presenting my argument, I do not claim that S&W’s view should be rejected in full. Both their view and the one presented here are incomplete views of moral responsibility. In the last section, I suggest some ways in which taking into account an agent’s control over her mental life can make a difference for practical purposes.

## A Case and Views

Consider the following case:

*Manipulated Miser*: Generous Jane has devoted her life to alleviating poverty, and wholeheartedly endorses that commitment. One day, Jane enters the hospital for surgery. While she is under anesthesia, a neurosurgeon secretly implants a new set of first- and second-order desires. Jane awakes with a miserly disposition. She finds her previous generosity misguided, desires to deny the needy her assistance, and wholeheartedly endorses that desire. [2: 177]

This case involves a radical change in at least one aspect of the agent’s moral character. Not only is this agent now miserly, she also fully supports this trait, due to the manipulation. As S&W suggest, “many will have the intuition that Jane’s responsibility for her post-operative miserly actions is substantially reduced” [2: 177]. But, of course, some agents can be fully responsible for acting in similar miserly ways. The challenge for views of moral responsibility is to point out a difference between normal agents that are fully responsible for their miserly actions and someone like Jane; a difference that can explain why Jane is less responsible.

Proponents of historical views of moral responsibility have sought to offer such an explanation by adding a historical component to their views, though they have mostly focused on cases where the agent is, intuitively, not responsible for the action at all. This component tends to take the form of a necessary condition on responsibility, requiring that, in order for an agent to be morally responsible for some action, she needs to have had (or lacked) a certain type of history. Although such a condition on responsibility is intended to capture the problematic feature of manipulation cases, it is only intended to be a part of an account of responsibility; it is not intended to provide a full account of responsibility. A full view of responsibility will need to take more things into account; e.g., the agent’s knowledge of, or culpable ignorance of, the relevant moral features of a situation, or the agent’s control at the time of action.

On one type of historical view, a *manipulator-focused view*, the explanation of why the manipulated agent is not responsible for her action essentially involves the presence of another agent intervening in the victim’s mental life in some way [6–9].^2^ On a different type of view, an *agent-focused view*, the explanation of the agent’s lack of responsibility makes no appeal to the presence of a manipulator. Rather, the explanation focuses on the history of the pro-attitudes leading to action [10–13]. Typically, these explanations appeal, at least in part, to the fact that these pro-attitudes were produced in a way that bypassed the agent’s capacities for control over her mental life. We can call this type of agent-focused view a *bypassing view*. On such a view, a crucial difference between a normal agent that is fully responsible for some action, and a manipulated agent that is not, can be found in the history of the pro-attitudes leading to the action; namely, the process by which they were produced.

As S&W point out, blind force cases can help motivate an agent-focused view. Blind force cases are like manipulation cases, but with the manipulator replaced by a natural force. For instance, we can suppose that Jane underwent this change not because of some meddling neurosurgeon, but rather because she passed “through a strange, electromagnetic field at the center of the Bermuda Triangle” [12: 168]. A manipulator-focused explanation will fail in this case, given that there is no manipulator present. An agent-focused view, on the other hand, may have an explanation available. A bypassing view, for instance, can offer an explanation similar to the one offered in the original case of *Manipulated Miser*, with the fact that the agent’s capacities for control over her mental life were bypassed playing a prominent role in the explanation. Blind force cases give us reason to develop an agent-focused view.

## The History-Sensitive Reflection View and Manipulation

Using parts of Christman’s view of autonomy [14–17], Sharp and Wasserman offer their own agent-focused partial view of moral responsibility that can help to explain why responsibility is diminished in cases like that of *Manipulated Miser*. The view, dubbed the *history-sensitive reflection view* (HSRV), states that:

An agent is fully morally responsible for actions issuing from some psychological characteristic C only if she is competent – she is, for example, capable of critical reflection, self-control and reasons-responsiveness – and she meets the following hypothetical reflection condition:

1. Were the person to engage in sustained critical reflection over a variety of conditions in light of the historical processes (adequately described) that gave rise to C,
2. She would not be alienated from C in the sense of feeling and judging that C cannot be sustained as part of an acceptable autobiographical narrative organized by her diachronic practical identity; and
3. The reflection being imagined is not constrained by reflection-distorting factors [2: 179–80]

S&W’s view of responsibility is incomplete in two main respects that will be relevant for the ensuing discussion. First, the condition is only concerned with *full* moral responsibility, by which is meant the degree of responsibility enjoyed by typical adults [2: 175]; it does not give us an account of what it takes to be morally responsible simpliciter. Second, HSRV only gives us a *requirement* on full responsibility, it does not give us a sufficient condition for full responsibility.^3^

Although HSRV is only a partial view, it may be enough to get us the claim that Jane is not fully responsible for her miserly actions, were we to further fill out the details. Suppose that, were Jane to undergo the process mentioned in HSRV, she would feel alienated from her miserliness. In this case, Jane would not be fully responsible for her post-manipulation miserly actions. This is so even if she does not actually undergo this process. An important aspect of full responsibility, on this view, is that it depends on reflections the manipulated agent performs counterfactually.^4^ Whether an agent meets HSRV will depend on whether the agent would come to a certain judgment, were she to reflect under certain conditions. We are not given much detail about what these conditions are, yet we are told their purpose. What is important for meeting HSRV is that the agent would not feel alienated over a variety of conditions, and “[t]his invariance is meant to establish that the self-acceptance in question is not idiosyncratic or fleeting” [17: 145].

Jane, in the actual world, does not feel alienated from her implanted miserly desires due to the fact that further second-order attitudes regarding her miserly desires have been implanted. In the case where she would feel alienated from these desires, were she to undergo the various reflections mentioned in HSRV, this would be because in these hypothetical reflections, she would have learned about the sources of these desires, and she would presumably have a strong aversion to this way of producing desires.

Given that the fulfilment of this condition relies on judgments counterfactually made by the agents, one might wonder whether a manipulator could simply extend his manipulation in order to ensure the right judgments in these counterfactual reflections. Specifically, one might wonder whether the manipulator could eliminate the aversion to these ways of producing desires. S&W consider a counterexample of this sort, a variation of *Manipulated Miser*. On this variation, Jane is further manipulated so that she would “not repudiate her miserliness, no matter what information about her history came to light” [2: 180]. S&W argue that this fails as a counterexample to HSRV. In this case, Jane’s capacity for critical reflection, and so her competence, has arguably been impaired: “[s]he now lacks the capacity to repudiate her manipulated actions upon reflection, a capacity essential to adequate reflection and so to her status *qua* competent agent” [2: 180]. Moreover, given her lack of competence, Jane’s reflection on her implanted trait would arguably be the result of “distorting factors that guarantee that the self-appraisal in question has a particular result” [16: 203].

I propose a different case which is a similar modification of *Manipulated Miser*. In this version, Sadie (Jane’s counterpart) is not manipulated such that she would “not repudiate her miserliness, no matter what information about her history came to light.” Sadie retains her capacity to repudiate her miserliness (even on the basis of reasons regarding the source of the miserliness), and in part, this is because she retains *some* aversion to the way that these desires were produced. Yet, this aversion has been weakened, such that she would not feel alienated from her miserliness were she to be placed in the counterfactual scenarios relevant to HSRV.^5^ Sadie, as S&W say of a different manipulated agent, “might resent the modification itself...but nevertheless find the outcome congenial” [2: 182].^6^ In fact, we do not need an attitude as strong as finding the manipulation congenial, we just need it to be the case that Sadie would not feel alienated from her miserliness. It is not clear that the change would lead to a change in our judgments about Sadie’s responsibility for her miserly actions; it still seems that she is not fully responsible. If one needs further motivation, consider the case of Beth, from the introduction. Suppose that although Beth would resent the fact that she was manipulated were she to be made aware of the intervention, she would not feel alienated from her desire to kill her neighbor. Beth also seems, at least, not fully responsible for her action.^7^ But HSRV cannot account for Sadie and Beth’s, at least, mitigated responsibility.

One might worry that in these cases, Sadie and Beth would still have their counterfactual reflection constrained by reflection-distorting factors, but it is not clear what these factors would be. This factor would not be the inability to repudiate their implanted traits, since they have this ability, they just would not repudiate them in the worlds relevant for the counterfactuals in HSRV. Further, once the manipulators have done their work in modifying the victims’ brains, they do no further work, and would not interfere in the process of reflection as it occurred. Moreover, in terms of the capacities of an agent that ground the ability to properly reflect, Beth and Sadie, by stipulation, have them to the same extent as agents who are fully responsible for similar actions. Because of these facts, it is difficult to see how Beth and Sadie’s counterfactual reflections would be constrained by reflection-distorting factors that normal, fully responsible, agents would not be subject to as well. A tempting idea is that some of the mental states used in the counterfactual process of reflection were implanted with the intention of the reflection’s yielding a certain result. The problem with taking this line is that it makes an appeal to an intention, and thus another agent. By doing so, the explanation falters in blind force variations of Beth and Sadie in which the changes are the result of natural forces, cases which include neither the second agent nor the intention.

Sadie and Beth both meet HSRV. Consequently, the view fails to account for the claim that these agents are, at least, substantially less responsible than normal agents who have not undergone a similar change. As far as HSRV tells us, they can still be fully responsible for these actions, yet this condition was intended to prevent this result. The problematic features of these cases, I argue, are more adequately captured by bypassing views.

## Bypassing and Control

In contrast to HSRV, bypassing views take the agent’s control over her mental life into account. What makes a historical view a bypassing one, as I use the term, is that part of the explanation for an agent’s lack of, or lessened, responsibility in these cases has to do with the fact that the attitudes leading to the action were produced in a way that bypassed the agent’s capacities for control over her mental life. Following S&W, I call these capacities the agent’s capacities for rational control. Bypassing views differ on various details, and in part, this is because the historical conditions tend to be part of a larger account of responsibility in general: terms of art employed in the proponent’s general account of responsibility are then used to state the historical condition. For brevity’s sake, I focus on Alfred Mele’s bypassing condition. This condition is compatible with most general accounts of moral responsibility, and requires the least amount of introduction. Mele does not offer a general account of moral responsibility, although he offers two independently necessary historical conditions for an agent’s responsibility for an action, intended to handle some extreme cases of manipulation.

Typically, a morally responsible agent will have, to some degree, capacities for rational control. Agents with these capacities are, for example:

capable of modifying the strengths of their desires in the service of their normative judgments, of bringing their emotions into line with relevant judgments, and of mastering motivation that threatens (sometimes via the biasing of practical or theoretical reasoning) to produce or sustain beliefs in ways that would violate their principles for belief-acquisition and belief-retention. They also are capable of rationally assessing their values and principles, of identifying with their values and principles on the basis of informed, critical reflection, and of modifying their values and principles, should they judge that to be in order. [13: 166-7]

A morally responsible agent will typically have these capacities, yet having them will not suffice for responsibility. For instance, without changing any details, we can stipulate that Beth has these general capacities to some extent when she acts soon after the manipulation. If one thinks that Beth is not responsible for the murder, then one has reason to think that having these capacities is not sufficient for responsibility.

One thing that different bypassing views agree on is that a problematic feature of these extreme manipulation cases is that these capacities were all bypassed in the production of the pro-attitudes that led to the action; these attitudes were produced in a way that did not engage with these capacities. Because of this, the agent was out of the control loop when it came to acquiring them. Of course, this is not all there is to the story. It is plausible that we attain many pro-attitudes in such a way, yet this does not eliminate our responsibility for actions issuing from these attitudes. On Mele’s view, although bypassing will not, by itself, undermine responsibility for actions that are the result of it, it can when it occurs in a certain way.

One important feature of cases of extreme manipulation is the degree of the change that occurred. In virtually all of the cases of extreme manipulation, the victim is turned into someone with a very different character than she previously had, at least with respect to some sphere(s) of her life. This degree of change, on its own, may not be enough to subvert responsibility for actions that flow from such a character. Someone may arrange to be the subject of such an intervention, for example, and this may be enough to make the agent responsible for some actions that are the result of the change. This is where bypassing can make a difference. The interventions in these cases occur without the agent’s knowledge or consent, and the new pro-attitudes are implanted in a way that bypasses the agent’s capacities for rational control. Had the agent been knowledgeable about the intervention and consented to it prior to the manipulation, then the new states would not have been produced in a way that fully bypassed these capacities. Were the change to be of a certain sort, as well as a result of bypassing, then it will at least mitigate moral responsibility for actions that are a result of the change.

Mele’s most recent historical condition focuses on the possibility of an agent who has suffered a radical change but still has “the greatest self-transforming powers that actual humans have” at (or shortly before) the time of action [1: 174]. Roughly, the condition states that an agent with these powers will not be responsible for some action that issues from values that were very recently implanted through bypassing if the action is of a type that her previous, long-standing, set of values were such that they would have prevented her from even acquiring a desire to perform this action, she was as responsible for having this previous set of values as any actual human can be, and either she cannot intentionally do otherwise than perform this action at this time, or if she can, the alternative would also be a product of implanted values that were a result of bypassing and that clash with her previous scheme.^8^

This condition is somewhat elaborate, and will not tell yield a verdict in every case of manipulation. This is because Mele’s main concern is with arguing that moral responsibility is a historical phenomenon, and only intends to capture cases where it is intuitive that the manipulated agent is not responsible.^9^ In order to meet this condition, the change the agent undergoes needs to be radical, at least with respect to this sphere of her life. Even though it is limited, it can still tell us something about the cases of Beth and Sadie. Supposing that, at the time of action (or shortly before), either Beth could not have intentionally done otherwise or were she to be able to, it would also be a product of other implanted attitudes, Beth meets this condition. Similar points apply to Sadie for miserly actions that fulfill the condition.^10^ These sorts of cases help to show that the notion of bypassing an agent’s capacities for rational control has an important role to play in a historical view of moral responsibility. On bypassing views, and unlike HSRV, whether these capacities for rational control were bypassed can play an important role regardless of whether the agent, as a result of further manipulation, would not feel alienated from the implanted attitudes.

S&W offer a brief objection to such views, and argue that HSRV has an advantage over them. A problem with other historical views, S&W suggest, is that the views are too demanding: “[f]or example, on Mele’s account, the mere fact that an agent acquired a certain psychological attitude in a way that bypassed her capacities for rational control is sufficient for denying her responsibility for actions that issue from that attitude” [2: 181]. By producing a condition on responsibility that is “fully external” to the agent, the views ignore the relevance of the agent’s evaluative scheme to responsibility; whereas HSRV accounts for this relevance.

There are a few points to make in response. First, a bypassing view need not be committed to the claim that acting from an attitude that was produced through bypassing is sufficient to undermine responsibility for that action. Mele’s own view, as we have seen, is more complicated, and he explicitly rejects this thesis [12: 148, 158, 13: 167].^11^

Further, it is not straightforwardly true that other historical views, including bypassing views, are “fully” external to the agent, or that they do not take the agent’s evaluative scheme to be relevant. For this point, it is helpful to distinguish between the agent’s evaluative schemes at different times. On HSRV, an agent’s evaluative scheme at the time of action, or just before, is of great relevance to full responsibility. The second-order attitudes that an agent has at this time – attitudes which are components of the evaluative scheme – will be one of the main determining factors in the results of the agent’s counterfactual reflections. On bypassing views, this is not the only time at which the evaluative scheme is relevant. Manipulation cases, including the case of Sadie, are meant to show that the agent’s evaluative scheme at, or just before, the time of action can be the result of further intervention. Insofar as we want to capture the problematic features of these cases, we will need to look elsewhere.

Now consider, instead, the agent’s evaluative scheme just before the agent is manipulated. The components of this scheme will be critical to the existence, and exercise, of the agent’s capacities for rational control. Recall, for instance, two of the capacities for rational control mentioned above: the capacity to identify with one’s values and principles on the basis of informed, critical reflection, and of modifying one’s values and principles, should one judge that to be in order. The exercise of these capacities will involve components of the agent’s evaluative scheme. When an agent employs these capacities to evaluate a new attitude, or a method that will lead to a change in attitudes, she will make use of the components of her evaluative scheme, including her values, desires, etc. On bypassing views, a problematic feature of cases like that of Beth and Sadie is that these capacities were bypassed at the time of manipulation; the agents’ evaluative schemes were not engaged by the manipulation. Notice that such an explanation still appeals to the agent’s evaluative scheme, and appeals to facts that are internal to the agent.

Although it is false that bypassing views place conditions that are “fully external” to the agent, or that they do not take the agent’s evaluative scheme to be relevant to her responsibility, it is true that these historical conditions do not tend to make reference to the evaluative scheme that the agent has at the time of action (or just before). In this connection, recall that historical conditions on responsibility are typically intended to provide a component of a view, not a complete view. Although historical conditions offer a requirement that an agent should meet in order to be responsible for an action, such conditions do not also state that meeting this requirement is enough to be responsible for that action. The fact that a historical condition ignores the relevance of an agent’s evaluative scheme at the time of action (or shortly before) does not imply that a full view incorporating this historical condition will be guilty of the same omission. Whether an agent actually (or hypothetically) endorses, or feels alienated from, the attitudes leading to an action will likely be relevant for responsibility, and will likely help to explain degrees of responsibility.^12^ If this is right, then the correct full view of moral responsibility will likely need to take the agent’s evaluative scheme (at, or just before, the time of action) into account. Whether the historical condition *in particular* should incorporate this evaluative scheme depends on whether doing so results in a condition that best captures the problematic features of manipulation cases.

One way in which bypassing views *could* take the agent’s post-manipulation evaluative scheme to be relevant is in explaining how an agent could return to full responsibility for actions that are the result of implanted traits. Proponents of bypassing views have not tended to focus on this question, and there is much to do in order to provide a clear and comprehensive answer. For now, I can gesture at an answer.^13^ I suggest that the main way for an agent to regain responsibility, or to return to full responsibility, will be through the exercise of her capacities for rational control. Were Sadie, after the manipulation, to end up accepting her miserliness after assessing it through the exercise of these capacities and engagement with attitudes that she held prior to the manipulation, she could become as responsible as a typical miserly agent for her miserly actions.

## Practical Questions

So far, I have argued that the correct historical condition will take into account an agent’s control over her mental life. In this section, I offer some brief thoughts on the practical implications of this discussion. Assuming that we have a theory of responsibility, discovering facts relevant to an agent’s responsibility will still be difficult in real-life cases. In recognition of this, S&W offer some rules of thumb for judging whether an agent meets HSRV. For instance, although the abruptness of a change will not be what mitigates an agent’s responsibility for actions that are the result of the change, if a change was particularly abrupt, then it is likely that the agent would feel alienated from such a trait after proper reflection [2: 183]. Another rule of thumb concerns whether the individual consented to, or knowingly and voluntarily risked, the change; if she did not, then she will be less likely to accept the change.

Incorporating a bypassing condition will likely change the strength of some of this evidence, particularly in cases where the agent did not consent to the change. Depending on the type of change under consideration, an agent’s lack of consent will not merely make it more likely that the agent would repudiate the results of the change, it would be constitutive of the agent’s, at least, lessened responsibility if the change is of the right sort. Thus, evidence of the agent’s lack of consent to such a change would provide greater justification for the claim that she is not fully responsible.

Adding a bypassing condition will also weaken the weights of certain pieces of evidence, given that there is more to take into account. Suppose that we have fairly good evidence that an agent meets HSRV with regard to some trait she gained due to an operation. Depending on how it is developed, a bypassing condition may weaken the strength of such evidence for the claim that the agent is fully responsible for actions issuing from such a trait. Even if a view takes counterfactual lack of alienation (in the right conditions) to be relevant to the degree to which an agent is responsible, the correct bypassing view will weaken the influence of this on the degree of responsibility if such lack of alienation would itself be due to further attitudes implanted by means of bypassing.^14^

## Conclusion

The goal of this paper has been modest. Appealing to historical views in the theoretical debate on moral responsibility is of great value, and can help to illuminate complex ethical issues concerning various ailments and treatments, including Deep Brain Stimulation. This paper joins Sharp and Wasserman in this endeavor. HSRV incorporates some aspects of responsibility that will likely be an important part of a full view of responsibility, and may play an important role in a historical condition itself. The main point of this paper is not to reject HSRV in full, rather it is to argue that the correct historical view will also incorporate an agent’s control over her mental life. Doing so can make the view capable of accounting for agents like Sadie and Beth. Although it is argued that the correct view will take this control into account, a complete view is not offered, and much work is yet to be done. Historical views have made advances in explaining why subjects of extreme manipulation may not be responsible for some actions, yet these views need to be developed in order to apply to more realistic, and less extreme, cases involving similarly worrisome changes. Particularly difficult will be figuring out which specific features of such interventions affect the degree to which an agent is responsible, and how much.

To conclude, I offer one final thought about the practical significance of getting this right. Recall the point made in the beginning about blind-force cases. Agent-focused views – which explain problematic cases of manipulation without mention of a manipulator – have the advantage that they can explain what is problematic about non-manipulation cases that raise similar worries; cases in which a change in the agent is due to natural forces. Not only does this provide agent-focused views with a theoretical advantage in terms of explanatory power, it also means that agent-focused views have a wider range of cases to which they apply. These views are useful in assessing the responsibility of agents who have undergone significant changes as a result of another agent’s intervention, such as some rare cases of DBS subjects, or, for instance, criminal offenders who have undergone chemical castration. But they are also useful in cases where the change is not the result of another agent’s actions, such as the famous case of Phineas Gage, or someone who, due to a brain tumor, has gained significant pedophilic tendencies [20].

## Footnotes

1 I intend “less extreme” to be understood broadly. Some ways, for example, for the manipulation to be less extreme is if it involves the implantation of a smaller amount of pro-attitudes, or did not erase competing pro-attitudes, or if the pro-attitudes implanted did not include attitudes about other implanted attitudes, or if the attitudes had less strength (e.g., if the desires implanted were weaker).

2 Sometimes people speak of relational views, as S&W do. I take it that if a view is a manipulator-focused one, then it is a relational one, but the entailment need not go both ways.

3 Although these are important restrictions grounded in the condition, S&W seem to have a fuller account in mind, though it is not clear what the details of the rest of the account are. For example, we are told that on HSRV, an agent would not be responsible [2: 180] and that two other agents would be responsible [2: 182], two claims that go beyond the scope of the account, as stated.

4 I here use “counterfactually” in a way that is standard for discussion of counterfactual conditionals. Counterfactual conditionals like the ones in HSRV can be true even if the agent undergoes the appropriate reflection and does not feel alienated in the *actual* world.

5 In part 1 of the condition above, S&W specify 2 and 3 as applying to sustained critical reflection *over a variety of conditions*. It is not clear how they understand the capacity that the original Jane lacks (the capacity to repudiate her miserly actions), but it cannot be that a necessary condition on having this capacity is that, for at least one of the conditions mentioned in 1, were the agent to undergo the process of sustained critical reflection under this condition, she would repudiate C. If this were a necessary condition on the capacity (which itself is necessary for the competence requirement), then no agent could both meet the competence requirement and meet 1–3. By meeting this necessary condition, the agent would fail to meet 1–3, since she would repudiate the actions (or trait) on at least one condition. This result would undermine S&W’s project, making it impossible for anyone to be fully responsible. If it is impossible to be fully responsible, then we lose the class of agents in comparison to which the manipulated agents are *less* responsible.

6 Christman himself does not consider this type of case, yet he does suggest that it would be incompatible with autonomy: “[i]n addition to reflective non-alienation, autonomy demands that such reflection is not itself merely the result of manipulative forces” [17: 146]. HSRV reflects Christman’s attempt to avoid this type of problem, yet the case of Sadie shows this attempt to be insufficient.

7 Suppose that someone has the intuition that Jane in *Manipulated Miser* is not fully responsible, yet lacks the intuition that Sadie, and this more developed version of Beth, are not fully responsible. One might ask this person: if the manipulation found in *Manipulated Miser* is problematic for responsible agency, why would ramping up that manipulation eliminate the problem? Obviously this should not be sufficient to convince this sort of person, but an answer to it would be helpful in clarifying the sources of disagreement.

8 Mele’s other condition is equally detailed yet focuses on an agent’s ability to shed the values leading to action [18: 268–9]. He also offers sufficient conditions for free action with a historical component [13].

9 The final clause, which includes the disjunction concerning the agent’s ability to do otherwise, is not clearly relevant to cases of mitigated, yet not eliminated, responsibility.

10 If one thinks that Sadie and Beth *are* responsible, yet not fully responsible, one might be willing to accept a variation on Mele’s view which replaces “will not be responsible” above with “will not be *fully* responsible.” The main point remains: contra HSRV, bypassing is relevant.

11 Fischer and Ravizza, who offer a different bypassing view, do not claim this either. The only view that may be committed to this is Haji and Cuypers’s view for developed agents [8]. Although this view may be too demanding in this respect, it is stated in terms of the agent’s evaluative schemes and its constituents.

12 Notice that here, there is no mention of knowledge regarding the sources of the attitudes that the agent endorses or is alienated from, as required by HSRV. This more general statement can be accepted by the proponent of HSRV, proponents of other historical views, *and* by non-historicists.

13 The points I make here are along similar lines as that taken by Fischer and Ravizza [10: 235], and appealed to by S&W [2: 181].

14 For a discussion of a similar issue concerning the related notion of authenticity in cases of anorexia nervosa, see [19].
